# Corolla chirality does not contribute to directed pollen movement in *Hypericum perforatum* (Hypericaceae): mirror image pinwheel flowers function as radially symmetric flowers in pollination

**DOI:** 10.1002/ece3.2268

**Published:** 2016-06-26

**Authors:** Carolina Diller, Charles B. Fenster

**Affiliations:** ^1^ Department of Biology University of Maryland College Park Maryland 20742; ^2^ Department of Biology and Microbiology South Dakota State University Brookings South Dakota 57007

**Keywords:** Disassortative mating, enantiomorphic contort aestivation, floral symmetry, mirror image, pollination, rotational symmetry

## Abstract

Corolla chirality, the pinwheel arrangement of petals within a flower, is found throughout the core eudicots. In 15 families, different chiral type flowers (i.e., right or left rotated corolla) exist on the same plant, and this condition is referred to as unfixed/enantiomorphic corolla chirality. There are no investigations on the significance of unfixed floral chirality on directed pollen movement even though analogous mirror image floral designs, for example, enantiostyly, has evolved in response to selection to direct pollinator and pollen movement. Here, we examine the role of corolla chirality on directing pollen transfer, pollinator behavior, and its potential influence on disassortative mating. We quantified pollen transfer and pollinator behavior and movement for both right and left rotated flowers in two populations of *Hypericum perforatum*. In addition, we quantified the number of right and left rotated flowers at the individual level. Pollinators were indifferent to corolla chirality resulting in no difference in pollen deposition between right and left flowers. Corolla chirality had no effect on pollinator and pollen movement between and within chiral morphs. Unlike other mirror image floral designs, corolla chirality appears to play no role in promoting disassortative mating in this species.

## Introduction

Floral symmetry design plays a prominent role in plant mating system patterns (Barrett 2002), pollination systems (Faegri and van der Pijl 1979; Johnson and Steiner 2000; Fenster et al. 2004), pollen transfer efficiency (Gómez et al. 2006), and angiosperm diversification rates (Sargent 2004; van der Niet and Johnson 2012). Symmetry patterns are often associated with directed pollinator movement and consequently pollen movement. For example, bilateral symmetry is associated with predictable placement of pollen on a pollinators body while asymmetric mirror image flowers (enantiostyly) found on the same plant not only place pollen on specific parts of a pollinator's body but also direct pollen on to opposite sides of a bees' body, greatly reducing the opportunity for selfing through geitonogamy (Sprengel 1793; Barrett 2002; Jesson and Barrett 2002a, 2005; Fenster et al. 2009).

Another, and highly understudied, floral symmetry pattern is present in flowers with contort aestivation and results from the mutual covering of petal flanks in the flower bud (aestivation pattern). In contort aestivation, each petal overlaps only one of its neighbor petals (Schoute 1935; Scotland et al. 1994). According to how the petals overlap, the corollas rotate clockwise or counterclockwise (also known as left and right, respectively) and are chiral to each other (see Fig. 2 for further explanation on terminology) (Schoute 1935; Scotland et al. 1994; Endress 2001). Corolla chirality is visibly distinguishable when the aestivation pattern is still present after anthesis and mainly when individual petals are asymmetric conveying the corolla a pinwheel appearance (Endress 1999, 2001). Endress (2001) summarizes the phylogenetic distribution of contort flowers across the angiosperm clade and distinguishes unfixed species (both right and left flowers are found on the same individual) from fixed species (all individuals of that species exhibit only one floral form). Note that no species have been observed where individuals are fixed for either right or left flowers, for example, in no species, are some individuals left and the remaining individuals right in the same population. Endress (2012) also refers to unfixed contort floral morphology as enantiomorphic, but henceforth we refer to this condition as unfixed corolla chirality. Most fixed species are asterids and most unfixed species, such as *Hypericum*, are rosids (Endress 1999, 2001). Very little is known of the adaptive biology underlying unfixed corolla chirality, although it is present within eight taxonomic orders and fifteen families within the rosids (Endress 1999).

Similar to monomorphic enantiostyly (Todd 1882; Gao et al. 2006), most unfixed chiral species have a 1:1 ratio of flower morphs within individuals (Davis 1964; Davis and Ramanujacharyulu 1971; Diller and Fenster 2014). In addition, Diller and Fenster (2014) found that the corolla chirality of a flower in two neotropical *Hypericum* species, *H. irazuense and H. costaricense,* is independent of the chirality of its closest neighbor flower, indicating a random distribution of corolla types within an individual. Some monomorphic enantiostylous species, such as *Heteranthera mexicana,* also present a random distribution of morph types as in *H. irazuense and H. costaricense* (Jesson et al. 2003). Despite the similarities to monomorphic enantiostyly, we do not know whether chirality variation has a parallel influence on pollen movement.

Corolla chirality differs from monomorphic enantiostyly by not having a reciprocal stamen to pistil arrangement between flowers. While enantiostyly increases outcrossing by the differential placement of pollen on the pollinator resulting from the alternate deviation of the style and stamen among flowers on the same individuals (Jesson and Barrett 2002a), species with unfixed corolla chirality do not have reciprocal placement of anthers and stigmas. However, the reduction in geitonogamous self‐pollination could still result if pollinators behave differently on right and left flower resulting in differential pollen placement on the pollinator's body. Honeybees and bumblebees can distinguish flowers by differences such as location (Makino and Sakai 2007), corolla shape and size (Galen 1996; Galen and Cuba 2001) color (Schemske and Bradshaw 1999), scent (Cnaani et al. 2006), and symmetry (Giurfa et al. 1996; Gómez et al. 2006). Given this variety of sensory differences that honeybees and bumblebees respond to, it is conceivable that bumblebees may also respond to the direction of pinwheel rotation of flowers, which is exceptionally visible in *H. perforatum* due to their asymmetric petals. Thus, we investigate the question of whether corolla chirality leads to asymmetric pollen‐movement between right and left flowers similar to enantiostylous flowers.

Here, we test whether unfixed corolla chirality is adaptive by decreasing geitonogamy through imposed directionality on pollen and pollinator movement, which to our knowledge has not been previously examined. We performed this study on *Hypericum perforatum,* an invasive plant of North America, and specifically asked: Is pollinator behavior or pollen movement influenced by chirality type? In addition, given that corolla chirality is an understudied trait, we quantified the frequency and distribution of right and left flowers within individuals to link this study with other studies on chirality ratios for floral traits.

## Materials and Methods

### Site description

This study was conducted at Mountain Lake Biological Station, Virginia, from 21 June 2012 to 10 July 2012 using two populations, one along a roadside (37° 22′ 360″ N, 80° 31′ 533″ W) and the other by an artificial pond (37° 22′ 459″ N, 80° 31′ 350″ W), which we will refer to as population A and B, respectively.

### Species description

**Figure 1:**
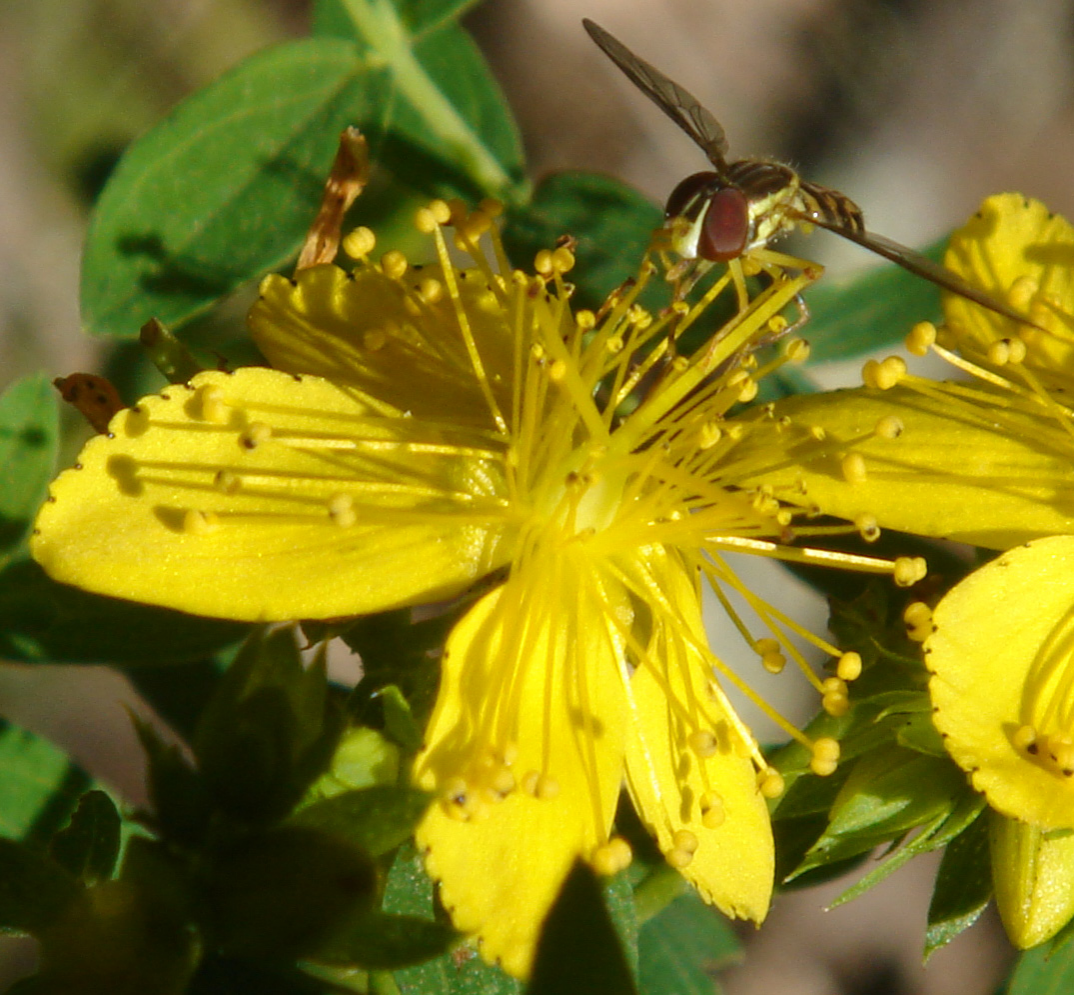
*Hypericum perforatum* flower with a syrphid fly (for scale) at the Mountain Lake Biological Station. This flower is an example of a right flower (see Fig. 2 for an explanation of terminology).

**Figure 2:**
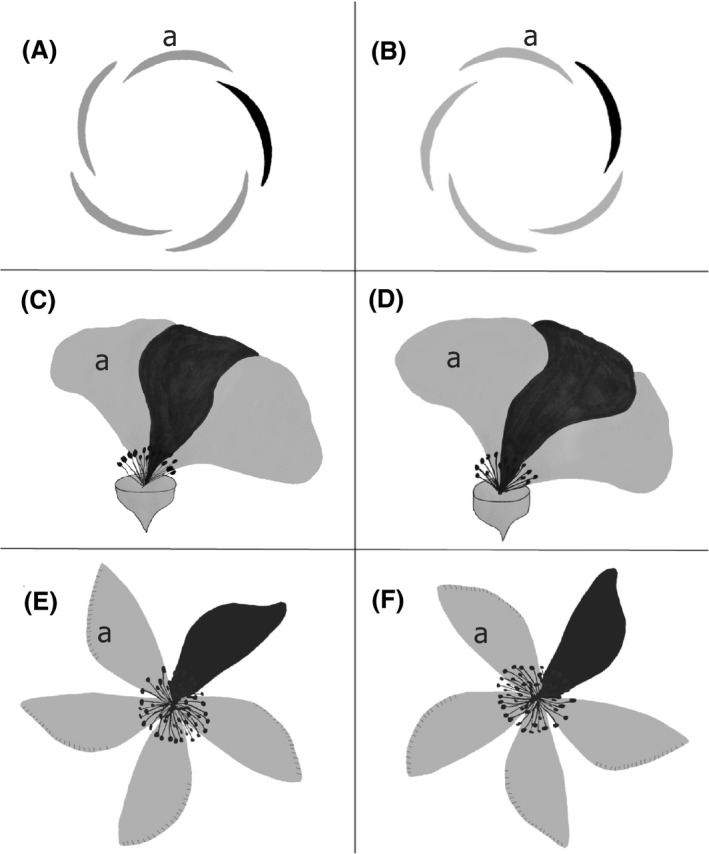
Explanation of the terminology of right and left chirality in *Hypericum perforatum* at the Mountain Lake Biological Station. Flower diagram at flower bud stage. (A) Left flower. Each petal overlaps its clockwise neighbor petal (e.g., petal “a” overlaps the shaded petal). Alternatively, if viewed from the side, the left of each petal overlaps its neighbor petal. (B) Right flower. Each petal overlaps its counter‐clockwise neighbor petal (e.g., the shaded petal overlaps petal “a”). Alternatively, if viewed from the side, the right of each petal overlaps its neighbor petal. Flower diagram at anthesis (open flower) (C) left flower (D) right flower. Simplified flower diagram (not to scale) at anthesis for *H. perforatum* (E and F). Overlap of petals is less evident in open flowers of *H. perforatum*. However, corolla chirality is still visually distinguishable due to petal asymmetry associated with chirality. Petals have one rounded and serrated side and one straight side. The rounded section defines the direction of the pinwheel rotation. (E) Left flower: pinwheel rotation is counterclockwise; (F) right flower: pinwheel rotation is clockwise. Right or left flowers are defined by the direction of overlap of petals, and not by the direction of the pinwheel rotation. Notice that the circular direction of the pinwheel and the overlap of petals in *H. perforatum* are opposite, that is, flowers with petals that overlap in a clockwise direction have a counter‐clockwise rotating pinwheel. However, both are “left” flowers, thus to avoid confusion in terminology referencing to petal overlap or corolla (pinwheel) rotation, here we define chiral morphs by right and left flower instead of clockwise and counterclockwise.

*Hypericum perforatum* L. (Hypericaceae) is a perennial shrub with flowers in a thyrse inflorescence, having five petals, three carpels, three stigmas, and numerous stamens (Fig. 1) (Crompton et al. 1988; Stevens 2007). In addition, every individual has two floral chiral types. These flowers can be easily distinguished by the shape and direction of their petals. Each petal is asymmetric with one straight side while the other is rounded and serrated. When an open flower is looked at from above, the right morphotype have the rounded side of every petal located on the right (Fig. 2F) and flowers of the left morphotype have it located to the left of every petal (Fig. 2E). *Hypericum perforatum* plants are typically 0.3–0.9 m tall (Crompton et al. 1988), and the mean number of open flowers per individual in our study site was 5.05 with a range of 1–15 flowers in population A (*n* = 22 individuals) and 8.53 with a range of 1–29 flowers per individual in population B (*n* = 35 individuals).

*Hypericum perforatum* is native to Europe and has been present in the United States since 1793, given incomplete herbarium records or historical documents on the presence of the species (Muhlenberg 1793; Sampson and Parker 1930). It is self‐compatible (Molins et al. 2014) as well as a pseudogamous facultative apomict (Matzk et al. 2001; Barcaccia et al. 2006; Molins et al. 2014). The latter means that *H. perforatum* can reproduce both sexually or asexually. Asexual reproduction in *H. perforatum* is characterized by the formation of an embryo without meiotic reduction nor fertilization (apomixis) (Barcaccia et al. 2006). Fertilization is, however, usually still required for the endosperm formation (pseudogamy) (Barcaccia et al. 2006). Crompton et al. (1988) state that fruit derived from selfed or cross‐pollinated flowers developed equally well in the greenhouse. Our observations confirmed these results for population B, but seed production was significantly greater for flowers cross pollinated by hand versus that had been excluded from pollinators in population A (Appendix S1A). In addition, we found no differences between right or left flowers in seed production for plants that were either: (1) excluded from pollinators with bags, (2) open‐pollinated, or (3) cross‐pollinated by hand (Appendix S1C and D) as well as no differences in pollen and ovule number (Appendices S2 and S3), respectively.

Because our study focuses on potential differences between right or left flowers and whether unfixed chirality mediates nonrandom pollinator or pollen movement, our investigations should not be unduly affected by the fact that the species is a facultative apomict. Many flowering plant species are facultative selfers yet have floral morphologies associated with promoting precise pollination and outcrossing (e.g., Fenster and Marten‐Rodriguez 2007). Analogously it is reasonable to assume that there are traits that promote outcrossing and precise pollination in a facultative apomict such as *H. perforatum*. Finally, we detected pollen limitation in both populations (Appendix S1B). The presence of both pollen limitation and greater seed production for cross‐pollinated flowers versus flowers excluded by pollinator in at least one population may suggest strong selection for floral traits related to promote outcrossing in *H. perforatum* (Knight et al. 2005 and references therein).

### Data sampling

#### Distribution of chirality types

To quantify the ratio of right and left chiral flowers within individuals, we counted the number of left and right flowers for 55 individuals (22 individuals in population A and 35 individuals in population B).

#### Chirality, pollinator interactions, and pollen movement

##### Pollinator visitation and preference

To determine whether pollinators discriminate among chirality types, we observed pollinator visitation for 23.5 h with 19 video observations (9 in population A and 10 in population B). The video observations were performed on 15 different plants and on 41 right flowers and 43 left flowers. As we were not able to identify the pollinators to the species level in the video observations, we classified them into three groups corresponding to large bees, small bees, and syrphid flies (for species examples, see Table 1). These groups were formed with the assumption that the pollinator size and behavior are similar within a group and could potentially differ between groups. For each pollinator visit, we recorded the chirality of the flower visited. We captured nine pollinators throughout the study and identified them to the species level (Table 1).

**Table 1:** Pollinator species captured and identified while visiting *Hypericum perforatum* flowers at Mountain Lake Biological Station (MLBS), VA. For the analyses, we classified the pollinators into three groups corresponding to large bees, small bees, and syrphid flies because of the expectation that pollinator size and behavior are similar within a group and would likely differ between groups. This is not an exhaustive list of possible species diversity visiting *H. perforatum* at MLBS, but an example of the most common visitors

Pollinator groups	Species
Apidae (large bee)	*Bombus griseocollis*
	*Bombus impatiens*
	*Bombus perplexus*
	*Bombus* sp.
Colletidae + Halictidae (small bee)	*Augochlora pura*
	*Lassioglossum cressonii*
	*Lassioglossum viridatum*
	*Hylaeus affinis*
Syrphidae (syrphid flies)	Unidentified to species

##### Pollinator sequence: Movement between flowers

We evaluated whether pollinators moved between right or left flowers in a random or nonrandom pattern. We examined the same video observations as above and registered for each pollinator observed the sequence in which they visited the flowers that were recorded in our videos. Each video period viewed at least one left and one right flower, and most more than one (with an average of right* *= 2.38 and left* *= 2.46 flowers). As an example, if two left and one right flower(s) were observed, and if a bee first visited the left flower, next the right, and then a left flower again, then we would report this visitation bout as a left–right–left sequence. Again, we identified the pollinator to pollinator group (Table 1) and not to the species level.

##### Pollinator behavior: Movement within flowers

**Figure 3:**
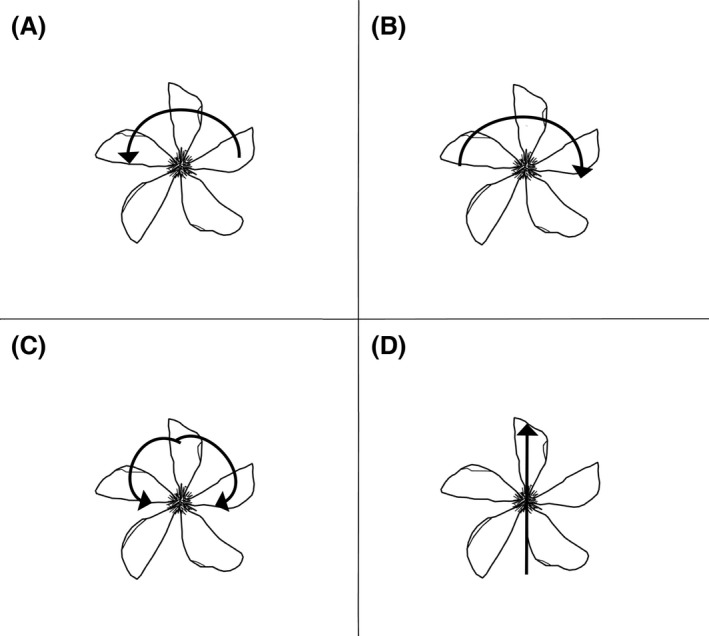
Pollinator behavior observed at the Mountain Lake Biological Station on *Hypericum perforatum*. Arrows indicate pollinator movement. (A) left rotation (B) right rotation (C) right and left rotation (pollinator rotates right, then turns and rotates left, or vice versa) (D) no rotation (pollinator starts at one side and travels in a straight line before leaving the flower).

We also examined how pollinators behaved when visiting a right and a left flower. With the same video observations as above, we registered how each pollinator moved during each floral visit. We identified four movement categories: right rotation, left rotation, both rotations, and no rotation (Fig. 3).

##### Pollen transfer

To evaluate the direction of pollen transfer from right and left flowers, we dyed the anthers of 14 flowers of one chirality type with a pink fluorescence powder during the morning. Later in the afternoon, nearby right and left flowers were collected from within a radius of 0.5 m of the dyed flower. All three stigmas were removed and checked under a fluorescent microscope for dye transfer, an analog of pollen movement (e.g., Fenster et al. 1996). Two days later, the experiment was repeated for the opposing chirality type at that same population. This 2‐day cycle was repeated five times. The period between experiments was implemented to avoid the carryover of fluorescence powder from the previous experiment with the opposite chirality type. During this experiment, we dyed a total of 140 flowers (70 right and 70 left flowers) and collected 138 left flowers and 139 right flowers on 19 individuals in population A and 18 individuals in population B.

#### Data analysis

All analyses were conducted with R: A Language and Environment for Statistical Computing (R Development Core Team, 2008) with nlme (Pinheiro et al. 2016), multcomp (Hothorn et al. 2008), and glmmADMB packages. We checked for the underlying assumptions to all statistical tests applied in this study and when the assumption was not met we transformed the data accordingly.

#### Distribution of chirality types

To determine whether the ratio of right and left flowers within an individual deviates from a 1:1 ratio, we calculated the proportion of left flowers for each individual and then compared the mean value (across all individuals) to 0.5 with a Student's *t*‐test. Observed proportions and the 0.5 expectation were arcsine square root‐transformed, and each individual was treated as a replicate for this test.

#### Chirality, pollinator interactions, and pollen movement

##### Pollinator visitation and preference

To evaluate whether pollinators prefer to visit each chirality type differentially, we performed a mixed model ANOVA (nlme package, R) and a post hoc Tukey test (multcomp package, R) on the visitation rates between right and left flowers. In this analysis, population and video observation were assigned as random factors (video observation nested within population) and chirality and pollinator group as a fixed factor.

In the analysis, each video observation represented a replicate. We calculated a standardized visitation rate for each video observation by:

$$\begin{array}{cc} & \frac{\sum \text{Right flowers visited}}{\sum (\text{Right flowers observed}\times \text{hours observed})}\;\\ & \text{and}\;\\ & \frac{\sum \text{Left flowers visited}}{\sum (\text{Left flowers observed}\times \text{hours observed})} \end{array}$$

In particular, for each video observation, we summed the number of right or left flowers visited and divided it to the total number of right or left flowers observed multiplied by the number of hours observed to generate a rate metric.

##### Pollinator sequence: Movement between flowers

To assess whether pollinators visit right and left flowers in a random sequence, we performed a mixed model ANOVA (nlme package, R) on the number of transitions that pollinators performed between right and left flowers. In this analysis, population and video observation were assigned as random factors (video observation nested within population) and chirality transition as a fixed factor. There were four possible chirality transitions: right‐to‐right (R‐R), right‐to‐left (R‐L), left‐to‐right (L‐R), and left‐to‐left (L‐L). The question addressed here is similar to the studies by Waser (1986) and Hopkins and Rausher (2012) on pollinator constancy or pollinator movement, but the analysis differs given that we did experimentally standardize for the number of right and left flowers to which pollinators were exposed to in each given video recording. To account for the fact that the videos differed in the number of right and left flowers observed as well as total number of visits, we calculated the deviation of the observed frequencies for each chirality transition from the expected, with the assumption that the pollinators move equally between right and left flowers. We calculated the expected frequencies by first calculating the expected proportion of flowers visited by each pollinator group in each video observation. For this, we multiplied the conditional probability of moving from one flower to the other by the probability of being on either a right or left flower. For further details and a worked through example please, see Appendix S4 and Table S1.

We constructed a parameter to measure an absolute deviation from expected random movement among chiral types for each camera. We subtracted the observed frequencies from expected frequencies (based on no bias of transition) and performed a square root transformation on the data. For this analysis, we eliminated videos for which less than 20 flower transitions were observed to assure that the deviations from the expected are not an artifact of low sample size. Thus, the range of flower transitions observed across all videos included in this analysis was 25–169 for the 11–15 camera observations, depending on the analyses. We asked whether there was a difference in transition probabilities among the four possible bee/flower transitions, that is, right‐to‐right, right‐to‐left, left‐to‐left, and left‐to‐right. That is, each of the transition sequences was treated as an independent replicate in our analyses.

##### Pollinator behavior: Movement within flowers

To test whether pollinators behave differently on right and left flowers, we performed a Generalized Linear Mixed Model (glmmADMB package, R) and a post hoc Tukey test (multcomp package, R). We fitted the data to a negative binomial distribution appropriate for our data and modeled pollinator behavior type (see Fig. 3) and chirality as fixed factors and population and video as random factors (with video nested within population). We tested the explanatory power of chirality and behavior by constructing a series of nested mixed models and comparing each model to the previous one using likelihood ratio tests. In addition, we chose to err on the side of the most conservative model by selecting a model with the least number of parameters within 3.22 units from the lowest AIC (Burnham and Anderson 2010; Fenster et al. 2015). For every video observation, we added the number of visits for each floral chirality and type of behavior combination observed. Then, we standardized these frequencies by dividing it by the number of flowers observed × hours of observation for each video. For example, two right and one left flower(s) were filmed for a period of 1.5 h during one video observation and a total of 24 pollinators visited the right flowers while 20 pollinators visited the left flower. In both cases, in this example, the pollinators moved without rotation (NR) while visiting the flowers. In order to calculate the standardized visitation rates for no rotation, both on a right and left flower, we divided 24 by two right flowers × 1.5 h and 20 by one left flower × 1.5 h, respectively. Each video observation was treated as a replicate for this test (*n* = 19). We also performed this analysis for large and small bees separately, but only report results for all pollinator combined due to no differences found among the main pollinator groups observed in this study.

##### Pollen transfer

Each stigma's fluorescence intensity was classified from a scale of one to five, with one being very little and five being very strong. Only one observer noted these measurements, reducing measurement error due to potential differences in the qualitative assessment of fluorescent intensity by different observers. The mean of the fluorescence intensity of the three stigmas was calculated for each flower. We used the fluorescence intensity as a proxy for pollen transfer (Kearns and Inouye 1993; Fenster et al. 1996) and performed a mixed model ANOVA (nlme package, R) to test potential differences in the quantity of pollen transfer and deposition between right and left flowers. In this analysis, population and block were random factors and donor (the chirality of the flower on which we applied the fluorescent dye on the anthers) and recipient (the chirality of the flower on which we qualitatively estimated the fluorescent dye on the stigmas) were fixed factors. We blocked our data every two consecutive experiment days (to control for climatic and other unexplained variation) resulting in five blocks. Each day we applied fluorescent dye to only one type of floral chirality; therefore, each block contains one right and left donor treatment. We standardized the mean fluorescence intensity for each flower with the highest fluorescence intensity of that given day to reduce the block interaction effect. Then, we averaged the fluorescence intensity for each individual within each block and performed an arc sine transformation in order to improve the normality of the data. Our replicate level was the average fluorescence intensity for each individual within each block, resulting in a total of 60 right donor individuals, 69 left donor individuals, 65 right recipient individuals, and 64 left recipient individuals in the analysis.

A significant donor effect indicates that right and left flowers donate pollen differentially, and a significant recipient effect indicates that right and left flowers are receiving pollen differentially. A significant interaction effect could demonstrate greater likelihood of transfer to opposite chirality.

## Results

### Distribution of chirality types

We observed 174 right flowers and 221 left flowers in both populations, 45 right and 60 left flowers in population A, and 129 right and 161 left flowers in population B. The proportion of left flowers within an individual differed marginally significantly from 0.5 in population B (mean = 0.64, 95% CI [0.44, 0.67], *t *=* *2.07, df = 33, *P *=* *0.05), but did not when both populations were combined as well as for population A (mean = 0.41, 95% CI [0.25, 0.59], *t *=* *0.99, df = 54, *P *=* *0.326; mean = 0.56, 95% CI [0.44, 0.67], *t *=* *0.99, df = 20, *P *=* *0.33, respectively).

### Chirality, pollinator interactions, and pollen movement

#### Pollinator visitation and preference

**Figure 4:**
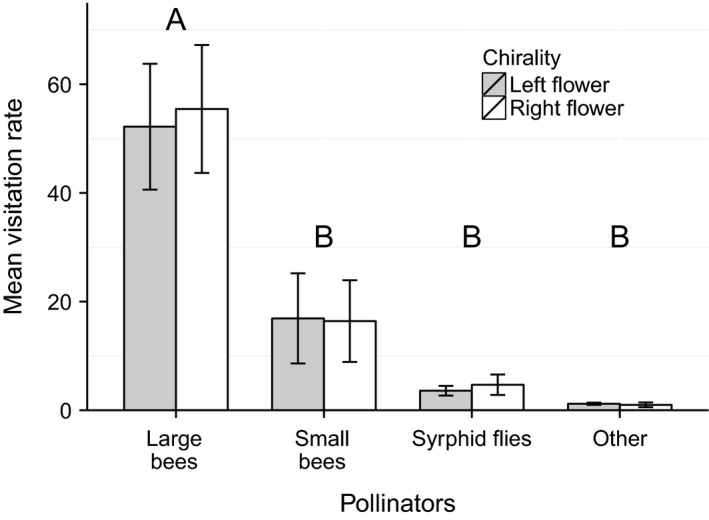
Pollinator visitation rate on right and left flowers of *Hypericum perforatum* as measured at the Mountain Lake Biological Station. Mean visitation rate (number of visits observed for right or left flowers/number of right or left flowers observed × hours of observation) for each pollinator group and chirality combination. The visitation rate was significantly different among pollinator groups (mixed ANOVA on all pollinator groups combined, *P *<* *0.001, *n* = 19 video observations) with the visitation rate for large bees statistically different to the other pollinator groups (Tukey, *P *<* *0.001, see “A” and “B” on graph). Pollinators did not discriminate between right and left flowers (mixed ANOVA on all pollinator groups combined, *P *=* *0.72). Error bars represent SE.

During the 23.5‐h video observations, the visitation rate was significantly different among pollinator groups (*F*_3, 76_ = 39.3, *P *<* *0.001, *n* = 19 video observations, see Fig. 4) with the visitation rate for large bees statistically different from the other pollinator groups (Tukey, *P *<* *0.001, see Fig. 4). However, there was no significant difference between the visitation rate for right (mean ± SE for the number of right flowers observed divided by the number of right flowers observed multiplied by hours of observation: 20.48 ± 2.47) and left (mean ± SE for the number of left flowers observed divided by the number of left flowers observed multiplied by hours of observation: 19.31 ± 2.82) flowers when all pollinator groups were combined (*F*_1, 76_ = 0.13, *P *=* *0.72, *n* = 19 video observations).

#### Pollinator sequence: Movement between flowers

Pollinators moved between right and left flowers at equal proportions. All deviations from the observed chirality transitions performed by the pollinators (i.e., right‐to‐right, right‐to‐left, left‐to‐right, and left‐to‐left), to the expected, did not differ significantly from one another (*F*_3, 42_ = 0.94, *P *=* *0.43, *n* = 15 video observations). The same was found when analyzing the two pollinator groups with the highest visitation rate separately: large bees (*F*_3, 42_ = 0.67, *P *=* *0.56, *n* = 15 video observations) and small bees (*F*_3, 30_ = 1.26, *P *=* *0.3, *n* = 11 video observations).

#### Pollinator behavior: Movement within flowers

**Figure 5:**
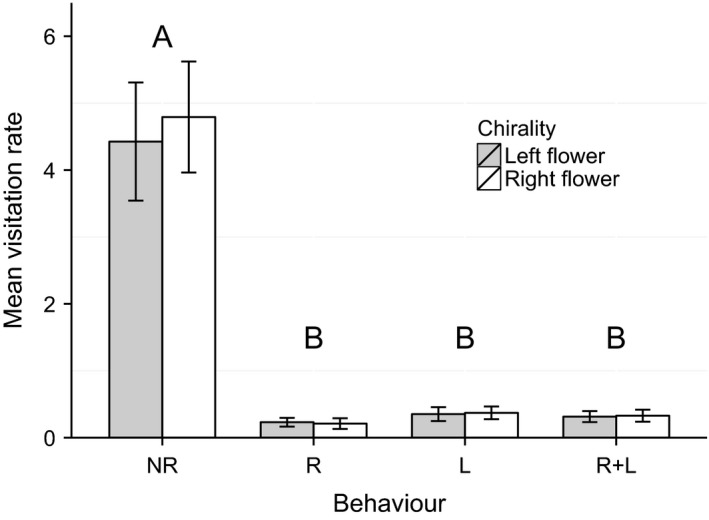
Pollinator behavior observed on right and left flowers of *Hypericum perforatum* at the Mountain Lake Biological Station. Mean visitation rates of all pollinators combined on right and left flowers with each of the following behavior types: NR = no rotation, L = left rotation, R = right rotation, and R+L = right and left rotation (see Fig. 3). “A” and “B” are significantly different from each other according to Tukey's test (*P *<* *0.001), indicating that pollinators preferred not to rotate (NR) while visiting a flower. There are no significant differences in pollinator behavior between right and left flowers (models that included chirality as an explanatory variable did not have a significantly lower AIC value). Error bars represent SE.

We found that pollinators do not adopt the four observed behaviors (rotate to the right “R”, to the left “L”, not rotate “NR”, or rotate to the left and right “R+L”; see Fig. 3) equally while visiting the flowers. The model with behavior type as a single fixed factor was the only one with a significantly lower AIC (likelihood ratio test; χ^2^ = 218.7, *P *<* *0.001; see Table 2). The post hoc Tukey test indicates a significant difference between no rotation (NR) and the rest of the pollinator behavior types (*P *<* *0.001, see Fig. 5), because of a significantly higher number of nonrotating (NR) visits to flowers. In addition, the post hoc Tukey's test shows no significant differences in pollinator behavior between right and left flowers (*P *=* *0.53–1; see Fig. 5).

**Table 2:** Model selection for pollinator behavior (movement within flowers) while visiting *Hypericum perforatum* flowers at Mountain Lake Biological Station, VA. Models in the table are arranged by increasing complexity starting with the null model. The null model includes the random factors, as intercept only models are not possible when fitting a generalized linear mixed model (glmmADMB package, R). Video is nested within population. “Test” indicates which models are tested in the likelihood ratio test

Model	Log likelihood	AIC	Test	χ^2^	*P* value
A. Null = Population + Video	−713.90	1437.8	–	–	–
B. Chirality + Population + Video	−713.71	1439.4	A versus B	0.392	0.5312
C. Behavior + Population + Video	−604.37	**1224.7**	A versus C	218.68	<0.001
D. Behavior + Chirality + Population + Video	−603.92	1225.8	D versus C	0.880	0.3482
E. Behavior + Chirality + Behavior × Chirality + Population + Video	−603.90	1231.8	E versus D	0.050	0.9971

χ^2^ and *P* values are outputs from the likelihood ratio test. The AIC value, in bold, indicates the model with the least number of parameters and within 3.22 units from the lowest AIC. Models were selected based on likelihood ratio tests and corroborated with AIC values.

#### Pollen transfer

There was no significant difference between the amount of fluorescence dye (analog of pollen) donated (*F*_1, 118_ = 1.26, *P *=* *0.26) or received (*F*_1, 118_ = 0.5, *P *=* *0.5) between right and left flowers.

## Discussion

This is the first study to explore whether corolla chirality imposes directionality on pollen movement. Differential pollen movement could occur if differences in corolla chirality prompt pollinators to interact differently on right and left flowers resulting in differential pollen placement on the pollinator's body. However, all of our observations indicate no effect of corolla chirality on pollen movement. Pollinators are indifferent to corolla chirality, and consequently there is no difference in pollen deposition between right and left flowers. Overall, we find no evidence that corolla chirality is an adaptation promoting precise pollination. We found a 1:1 ratio of left and right flowers within individuals, concordant with ratios found for other species with unfixed corolla chirality (Davis 1964; Davis and Ramanujacharyulu 1971; Diller and Fenster 2014).

Different patterns of pollinator movement on right and left flowers may translate into differential pollen placement on the pollinator's body, and thus an increase in pollen carry over distance and a reduction in geitonogamy. Our hypothesis was that pollinators may move clockwise or counterclockwise, depending on the rotational pattern of the flower, leading to different pollen placement on the body of the pollinator. Bilateral symmetric flowers are considered to increase pollinator directionality by forcing pollinators to approach the flower from only one direction and thus are thought to also increase pollen movement efficiency and directionality (Neal et al. 1998; Gómez et al. 2006). A comparable process could result in *H. perforatum* flowers if, for instance, pollinators have an innate preference to move in a circular mode from the rounded side toward the straight side of the petal. In this example, pollinators would move right (clockwise) in the left flowers and left (counterclockwise) in the right flowers. Differential rotational movement while probing for pollen on the flower could potentially result in differential pollen placement on the right and left area of the pollinator's body. However, we find no evidence that unfixed chirality is associated with directed pollinator movement on the flower. Bees treat the different chiral types indiscriminately, mostly moving straight across the flower (no rotation), and when turning on the flower, their turns are unrelated to chiral type. In this sense, the interaction of *H. perforatum* with pollinators resembles that of typical vertically oriented, radially symmetric flowers, which are less capable of directing pollinator movement compared to bilateral flowers (Fenster et al. 2009).

Reciprocal herkogamy, such as heterostyly or enantiostyly, is one of the reproductive strategies that increase pollen movement in flowers with radially symmetric corollas (Barrett 2002). Heterostylous flowers have anthers and stigmas that differ reciprocally in position among flowers of different individuals and are usually associated with incompatibility between morph types. Pollen–stigma incompatibility between right and left flowers could have also contributed to pollen movement directionality in *H. perforatum* despite the lack of reciprocal positioning of anthers and stigmas. We find no pollen–stigma incompatibility between right and left flowers (Appendix S1E), but these results need to be interpreted with caution given that we did not emasculate the flowers, and because *H. perforatum* is a facultative apomict. Heterostyly evolved in three species of *Hypericum* that also have corolla chirality, but the incompatibility between stamen–pistil height morphs is not complete in the most studied species, *H. aegypticum* (Ornduff 1975). However, *H. aegypticum* does not have a strong petal asymmetry associated with corolla chirality, and more importantly to our study, the study makes no mention that the two heterostylous morphs correlate with a specific chirality type.

While studies are lacking to fully comprehend to what extent plant mirror image structures (both vegetative and reproductive) differ in their genetic control and developmental pathways, it seems that there is some variation in the degree to which they are genetically or environmentally determined. Cross experiments show no inheritance in the direction of leaf phyllotaxy (Allard 1946; Davis 1962; Hashimoto 2002), in contrast to mendelian genetic control for the expression of style orientation in the dimorphic enantiostylous *Heteranthera multiflora* (Jesson and Barrett 2002a,b) and the direction of twisting of pods for *Medicago turberculata* and *M. litoralis* (Lilienfeld and Kihara 1956). Additional studies successfully induced spiraled roots, stems, and flower organs in *Arabidospis thaliana* through mutations (see references in Hashimoto 2002). Relevant to the discussion of unfixed corolla chirality is that the direction of the helical growth can be either fixed or random depending on the genes disrupted (Hashimoto 2002). To our knowledge, there are no other studies on the genetic control for mirror structures when both morphotypes are expressed within an individual. Nevertheless, even if the chiral identity of a new shoot or a new flower bud is environmentally determined (such as by the symmetry of the entire inflorescence (Endress 1999, 2001)), this does not exclude the possibility for unfixed mirror image structures to be adaptive as in monomorphic enantiostyly.

Most of the examples of chirality in vegetative structures and other reproductive structures also challenge an adaptive explanation and instead suggest a neutral hypothesis (e.g., leaf phyllotaxy, stem twisting, cone spirality, fruit arrangement in sunflower heads, or circumnutation, that is, the helical movement of plant organs) (Allard 1946; Davis and Ramanujacharyulu 1971; Davis and Davis 1987; Minorsky 1998; Klar 2002; Edwards et al. 2007; Stolarz 2009). An exception to this, apart from enantiostyly, is anisophylly with dorsiventral shoot symmetry, that is, the pair of dorsal leaves is smaller than the pair of ventral leaves. This type of vegetative mirror image is thought to reduce leaf shade of dorsal leaves to the ventral leaves and thus increase photosynthetic surface area (Dengler 1999; Muelbert et al. 2010).

This study provides evidence that unfixed corolla chirality, unlike mirror image enantiostyly, does not represent an adaptation associated with promoting disassortative mating between floral morphs, or directed movement of pollen between flowers. Instead, our findings demonstrate that unfixed corolla chirality may be similar to other radial symmetrical flowers with open and generalized pollination system and consequently does not direct pollen movement between flowers. It remains to be determined whether these findings are generalizable to other major clades where corolla chirality is also found.

## Conflict of Interest

None declared.

## Supporting information


**Appendix S1.** Reproductive biology of *Hypericum perforatum* at MLBS.
**Appendix S2.** Chirality and pollen number.
**Appendix S3.** Chirality and ovule number.
**Appendix S4.** Pollinator sequence: Movement between flowers.

**Table S1.** Pollinator sequence: Movement between flowers.
