# Supplementary material for: Corolla chirality does not contribute to directed pollen movement in Hypericum perforatum (Hypericaceae): mirror image pinwheel flowers function as radially symmetric flowers in pollination
Source: Ecol Evol. 2016 Jun 26;6(14):5076–86. doi: 10.1002/ece3.2268 (PMC4979728; doi:10.1002/ece3.2268)
Supplement: Supplementary file 1 — Appendix S1. Reproductive biology of Hypericum perforatum at MLBS. Appendix S2. Chirality and pollen number. Appendix S3. Chirality and ovule number. Appendix S4. Pollinator sequence: Movement between flowers. [file ECE3-6-5076-s001.docx]

**Appendix S1.**

**Data sets for sections S1. A- E*—**

*We did not emasculate flowers in the three data sets described below and this should be taken into account when interpreting the results presented in sections appendix S1. A-E.

*Data set 1—* Flower buds were bagged for 13 individuals (6 from population A and 7 from population B). Once opened, a right and left flower was selected per individual whenever possible, and left bagged until the flowers senesced. We ended up with 5 left and 6 right flowers in population A and 7 left and 6 right flowers in population B, resulting in 12 left and right flowers in total.

*Data set 2—* We cross-pollinated two right and two left flowers on each individual. Each flower morph was once hand-pollinated with pollen from a right flower and once with pollen from a left flower to test for all donor–recipient chirality combinations. After pollinating the flowers we bagged them to assure that no additional pollen was deposited. This experimental design was replicated on 17 individuals (7 from population A and 10 from population B), resulting in 17 left flowers pollinated with another left flower (LXL), 17 left flowers pollinated with a right flower (LXR), 15 right flowers pollinated with a left flower (RXL) and 16 right flowers pollinated with another right flower (RXR).

Data set 3— For each individual, we added pollen on two right and two left flowers, whenever possible. Each floral chirality received pollen from both a right and left flower of a different

individual. In addition, we had two control flowers (a right and left) without pollen addition. All flowers had been previously bagged. This was replicated for thirteen individuals (6 from population A and 7 from population B), resulting in 12 left flowers with pollen supplementation from another left flower (LXL), 13 left flowers supplemented with pollen from a right flower (LXR), 13 right flowers supplemented with pollen from another right flower (RXR), 11 right flowers supplemented with pollen from a left flower (RXL), as well as 13 left and 13 right flowers as control treatments.

**S1. A. Seed production in *H. perforatum*- flowers excluded from pollinators vs flowers cross pollinated—** To determine whether cross pollination produces more seeds than seed produced either by selfing or facultative apomixes (flowers excluded from pollinators) we compared data set 1 and 2 independently of whether flowers were right or left. We averaged the number of seeds per individual. Because we found a significant interaction between population and treatment (i.e. excluded vs cross pollinated) (two way ANOVA (stats package, R) with population and treatment as fixed factors; *F*_1, 26_ = 4.56, *P* = 0.04) we performed a separate one way ANOVA (stats package, R) for each population. The average seed production per individual for flowers excluded vs cross pollinated treatments did not differ significantly for population B (excluded: mean ± SE = 53.75 ± 7.4, n = 7; cross pollinated: mean ± SE = 45.2 ± 6.3, n = 10; *F*_1, 15_ = 0.74, *P* = 0.4) but was marginally significant for population A (excluded: mean ± SE = 31.8 ± 7.6, n = 6; cross pollinated: mean ± SE = 54.1 ± 7, n = 7; *F*_1, 11_ = 0.471, *P* = 0.053).

**S1. B. Pollen limitation in *H. perforatum*—** To determine whether *H. perforatum* was pollen limited, we compared the seed production of flowers with pollen addition vs. control flowers (data set 3), independently of whether flowers were right or left. We averaged the number of seeds per individual. We found no significant interaction effect between population and treatment (i.e. pollen addition and control) (*F*_1, 22_ = 2.64, *P* = 0.12) and thus performed mixed model ANOVA (nlme package, R) with individual and population as random factors (individual nested within population) and treatment (pollen added vs control) as fixed factor on these averages. We found a marginally significant difference (*F*_1, 12_ = 4.66, *P* = 0.052) in the seed production between flowers with added pollen (48.5 ± 3.8, n = 13) and control flowers (38.6 ± 3.31, n = 13), indicating that flowers are pollen limited.

**S1. C. Chirality and pollinator exclusion—** We used data set 1 to determine whether seed production differed between right and left flowers when excluded from pollinators. Because our data set is unbalanced, we checked for an interaction effect between treatment (i.e. chirality) and population with a two way ANOVA (stats package, R) with population and treatment as fixed factors. No interaction was found (*F*_1, 20_ = 0.0008, *P* = 0.98) so we performed a mixed model ANOVA (nlme package, R) with individual and population as random factors (individual nested within population) and chirality as fixed factor**.** We found no significant difference in seed production between right and left flowers when they were excluded from pollinators (right flowers: mean ± SE = 38.8 ± 7.72, left flowers: 46.1 ± 5.73, *F*_1, 10_ = 1.797, *P* = 0.21).

**S1. D. Chirality and pollen limitation (open pollination vs cross pollination by hand)—**We used data set 3 to check whether right and left flower had differential pollen limitation. Here again, we checked for an interaction effect between treatment and population with a two way ANOVA (stats package, R) with population and treatment as fixed factors. When no interaction was found (*F*_5, 63_ = 1.2, *P* = 0.32) we performed two separate mixed model ANOVA (nlme package, R) with individual and population as random factors (individual nested within population) and treatment as fixed factor. Treatment had six levels: control/open pollination right flower, control/open pollination left flower, pollen added from left flower to right flower, pollen added from left flower to left flower, pollen added from right flower to left flower and pollen added from right flower to right flower. Right and left flowers showed no pollen limitation (*F*_5, 57_ = 1.62, *P* = 0.17). Flowers produced the same amount of seed independently of whether the pollen supplied was taken from a right or left flower.

**S1. E. Pollen incompatibility associated with chirality pollen donor - recipient combinations—**We used data set 2 to test for pollen-stigma incompatibility associated with chirality. We compared seed production between right and left flowers after cross pollinating them with various combinations of pollen from the two chirality types (i.e. left flowers pollinated with pollen from another left flower [LXL], left flower pollinated with a pollen coming from a right flower [LXR], right flowers pollinated with pollen coming from a left flower [RXL] and right flowers pollinated with pollen from another right flower [RXR]). When no interaction was found between population and treatment (*F*_3, 57_ = 0.376, *P* = 0.77) we performed a mixed model ANOVA (nlme package, R) with individual and population as random factors (individual nested

within population) and cross-pollination treatment combinations as a fixed factor. We found no significant difference whether the pollen donor or recipient was a left or right flower in the outcrossing treatments (*F*_3, 45_ = 0.71, *P* = 0.55). Flowers produced the same amount of seed independently of whether the pollen supplied was taken from a right or left flower (mean ± SE: right donor and recipient: 51.38 ± 6.6, right donor and left recipient: 48.8 ± 6.378, left donor and right recipient: 46.7 ± 5.72, left donor and recipient: 51.4 ± 5.01).

***Appendix S2. Chirality and pollen number****—*To determine whether right and left flowers differ in pollen number, we collected right and left flowers from 21 individuals (11 from population A and 10 from population B). These flowers were previously bagged flower buds and belonged to the same inflorescence. For each flower we selected three dehisced and untouched stamens and placed the stamens in a 1.5 ml Eppendorf tube in 0.1 ml of aniline blue dye. After mixing the solution with a pipette we took two samples of 10 microliters from the 0.1 ml pollen solution with aniline blue dye and quantified pollen production with a hemocytometer. We counted the pollen on the four 1 mm^2^ hemocytometer squares and calculated the mean of the two solution samples for each flower. We performed a mixed model ANOVA (nlme package, R) with population and individual as random factors (individual nested within population) and chirality as the fixed factor to compare the number of pollen per flower between right and left flowers. No significant differences were detected for pollen number between right (mean ± SE for pollen grains per anther: 160 ± 14.24) and left (mean ± SE for pollen grains per anther: 157 ± 15.93) pairs of flowers (*F*_1, 20_ = 0.025, *P* = 0.87).

***Appendix S3. Chirality and ovule number****—*To determine whether right and left flowers differ in ovule number we collected one right and left flower for each of 20 individuals (10 individuals from each population), dissected the three carpels and counted ovule number under a dissecting scope. For 8/20 individuals we collected adjacent right and left flowers, controlling for developmental timing. No differences were found between the two sampling methods (adjacent right and left flowers within an individual vs non adjacent flowers but still within the same individual), and so we report the combined data. We performed a mixed model ANOVA (nlme package, R) with population and individual as random factors (individual nested within population) and chirality as the fixed factor to compare the number of ovules per flower between right and left flowers. No significant differences were detected in ovule number between right (mean ± SE = 245 ± 7.80) and left (255.1 ± 6.67) flowers (*F*_1,19_ = 1.43, *P* = 0.25).

**Appendix S4. Pollinator sequence: Movement between flowers—**

Further explanation on how we calculated expected proportions for the analysis on pollinator sequence: If the video camera was set to observe four right flowers and two left flowers, the expected proportion given that the visit sequence is random would be:

|  | **Probability of being on either a Right (R) or Left flower (L)** | **Conditional**  **Probability of moving to the next flower of given chirality type** | **Final expected proportions** |
| --- | --- | --- | --- |
| R-R | 4/6 | 3/5 | 6/15 |
| R-L | 4/6 | 2/5 | 4/15 |
| L-R | 2/6 | 4/5 | 4/15 |
| L- L | 2/6 | 1/5 | 1/15 |

In the above example, if a bee is on a right chiral flower then there are only five flowers to which it can move and three of them are right, the pollinator has 3/5 probability to move to another right flower and 2/5 probability to visit a left flower. In addition, the probability of visiting a right flower in this given scenario of four right flowers and two left flowers is 2/3 (4 right flowers/6 total flowers). Therefore the final expected proportion of visitation sequences by a pollinator from a right chiral flower to another right chiral flower is 2/3 * 3/5 = 6/15. With these expected proportions we calculated expected frequencies, by multiplying the expected proportion with the total number of flower transitions made by a pollinator during that video observation. We were only interested in pollinators moving away from the flower and therefore did not include pollinators immediately returning to the same flower as a new visit.
